# A Knockout Mutation of a Constitutive GPCR in *Tetrahymena* Decreases Both G-Protein Activity and Chemoattraction

**DOI:** 10.1371/journal.pone.0028022

**Published:** 2011-11-29

**Authors:** Thomas J. Lampert, Kevin D. Coleman, Todd M. Hennessey

**Affiliations:** Department of Biological Sciences, University at Buffalo, Amherst, New York, United States of America; German Institute for Human Nutrition, Germany

## Abstract

Although G-protein coupled receptors (GPCRs) are a common element in many chemosensory transduction pathways in eukaryotic cells, no GPCR or regulated G-protein activity has yet been shown in any ciliate. To study the possible role for a GPCR in the chemoresponses of the ciliate *Tetrahymena*, we have generated a number of macronuclear gene knockouts of putative GPCRs found in the *Tetrahymena* Genome database. One of these knockout mutants, called G6, is a complete knockout of a gene that we call GPCR6 (TTHERM_00925490). Based on sequence comparisons, the Gpcr6p protein belongs to the Rhodopsin Family of GPCRs. Notably, Gpcr6p shares highest amino acid sequence homologies to GPCRs from *Paramecium* and several plants. One of the phenotypes of the G6 mutant is a decreased responsiveness to the depolarizing ions Ba^2+^ and K^+^, suggesting a decrease in basal excitability (decrease in Ca^2+^ channel activity). The other major phenotype of G6 is a loss of chemoattraction to lysophosphatidic acid (LPA) and proteose peptone (PP), two known chemoattractants in *Tetrahymena*. Using microsomal [^35^S]GTPγS binding assays, we found that wild-type (CU427) have a prominent basal G-protein activity. This activity is decreased to the same level by pertussis toxin (a G-protein inhibitor), addition of chemoattractants, or the G6 mutant. Since the basal G-protein activity is decreased by the GPCR6 knockout, it is likely that this gene codes for a constitutively active GPCR in *Tetrahymena*. We propose that chemoattractants like LPA and PP cause attraction in *Tetrahymena* by decreasing the basal G-protein stimulating activity of Gpcr6p. This leads to decreased excitability in wild-type and longer runs of smooth forward swimming (less interrupted by direction changes) towards the attractant. Therefore, these attractants may work as inverse agonists through the constitutively active Gpcr6p coupled to a pertussis-sensitive G-protein.

## Introduction

The ciliated protozoan *Tetrahymena thermophila* shows chemosensory responses to many different stimuli but no chemoreceptors have been fully verified from gene to ligand. As free-swimming cells, *Tetrahymena* change their swim speed and swimming direction in response to many types of chemorepellents [1], [2], [3], [4] and chemoattractants [5], [6], [7], [8]. These changes in swimming behaviors allow them to generate directed movement away from hazardous locations and towards preferred areas of their fresh water environment. The general model from studies of the related ciliate, *Paramecium*, is that chemoattractants cause somatic hyperpolarization, faster forward swimming speed, and less directional changes [9]. Chemorepellents in *Paramecium* cause depolarizations that elicit repetitive bouts of backwards and forwards swimming called “avoiding reactions “ (AR) by generating Ca^2+^-based action potentials and inward Ca^2+^ currents through the ciliary voltage-dependent Ca^2+^ channels [10]. As intraciliary free Ca^2+^ rises, the beat frequency slows and when the free Ca^2+^ exceeds 10^−6^ M, the cilia reverse their direction of beat [10], [11]. Therefore, these unicells integrate sensory information in the form of changes in membrane potentials to generate an appropriate ciliary response. The intracellular electrophysiological measurements in *Tetrahymena* have shown that they are generally similar to those of *Paramecium*, establishing *Tetrahymena* as a suitable tool for studies of membrane excitation and chemosensory transduction mechanisms [12], [13].

Many chemosensory reception systems in eukaryotic cells commonly start with ligand activation of a G-protein coupled receptor (GPCR) [14], [15]. GPCRs are seven-transmembrane spanning proteins that typically affect the function of a chemosensory transduction pathway through a change in the associated heterotrimeric G-protein activity [16] and they are predicted to be present throughout the majority of sequenced eukaryotic genomes [17]. Sensory cells from nematodes to vertebrates express hundreds of GPCR genes that play critical roles in both olfaction and gustation through heterotrimeric G-protein activation [18]. In yeast, GPCRs have been shown to play important roles in their nutrient and pheromone sensing pathways [19], [20]. *Dictyostelium* has also been shown to possess several GPCRs involved in chemotaxis, cellular aggregation, and sporulation [21].

Several studies have provided evidence supporting the hypothesis that canonical GPCRs are present in several ciliates but no GPCR or regulated G-protein activity has been described in any ciliate. Antibodies to homologous and cloned fragments have implied the existence of G-proteins in *Tetrahymena*, *Paramecium*, and *Stentor* [22], [23], [24]. Alterations in behavior have been reported by treatment with both PTX and CTX in several ciliates [6], [23], [24], [25]. PTX induced ADP-ribosylation of specific proteins has been demonstrated in *Paramecium* [23] and the distantly related fellow alveolate *Plasmodium* [23], [26]. It has also been suggested that the ciliary voltage-dependent Ca^2+^ channels in *Paramecium* are modulated by PTX sensitive G-proteins [23], [27]. This provides a possible link between constitutive GPCR activity and membrane excitability in the ciliates.

Although the original GPCR model was that agonists exert their effects on GPCRs that have little or no basal activities, there are now many GPCRs that have been shown to have constitutive activities in the absence of added ligands [28], [29]. In addition, constitutive GPCR activities have been shown to be modulated by inverse agonists, generating differential signals by decreasing basal G-protein activities [30], [31], [32]. Gene knockout techniques have provided great insights in the functions of GPCRs in many eukaryotic organisms [21], [33], [34]. Therefore, we have used this approach to study the functions of GPCRs in *Tetrahymena*.

It is important to note that *Tetrahymena* are different from many eukaryotic cells because they possess two distinct nuclei, a polyploid macronucleus and diploid micronuclei. The micronuclei are the sexual genetic repository of the cell and their DNA is not normally transcribed. The macronucleus is the somatic nucleus where genes are actively transcribed. As a result of conjugation (mating) of two different mating types, the old macronucleus is degraded and a new micronucleus is generated from the old micronuclear DNA. Therefore, there are two types of genetic knockouts in *Tetrahymena*, micronuclear [35] and macronuclear [36]. Micronuclear knockouts are made by introducing linearized knockout constructs into mating cells while macronuclear knockouts can be made from vegetative, non-mating cells. Micronuclear knockout heterokaryons require an additional sexual cycle between two different mutant mating types to create a homozygous complete knockout in the macronucleus and micronucleus. Since different mating types can show strikingly different behavioral phenotypes (personal observations), we have chosen to make macronuclear gene knockouts to provide a clear uniparental control.

## Results

### Characterization and amino acid sequence analysis of the putative GPCRs in *Tetrahymena thermophila*

**Figure 1:**
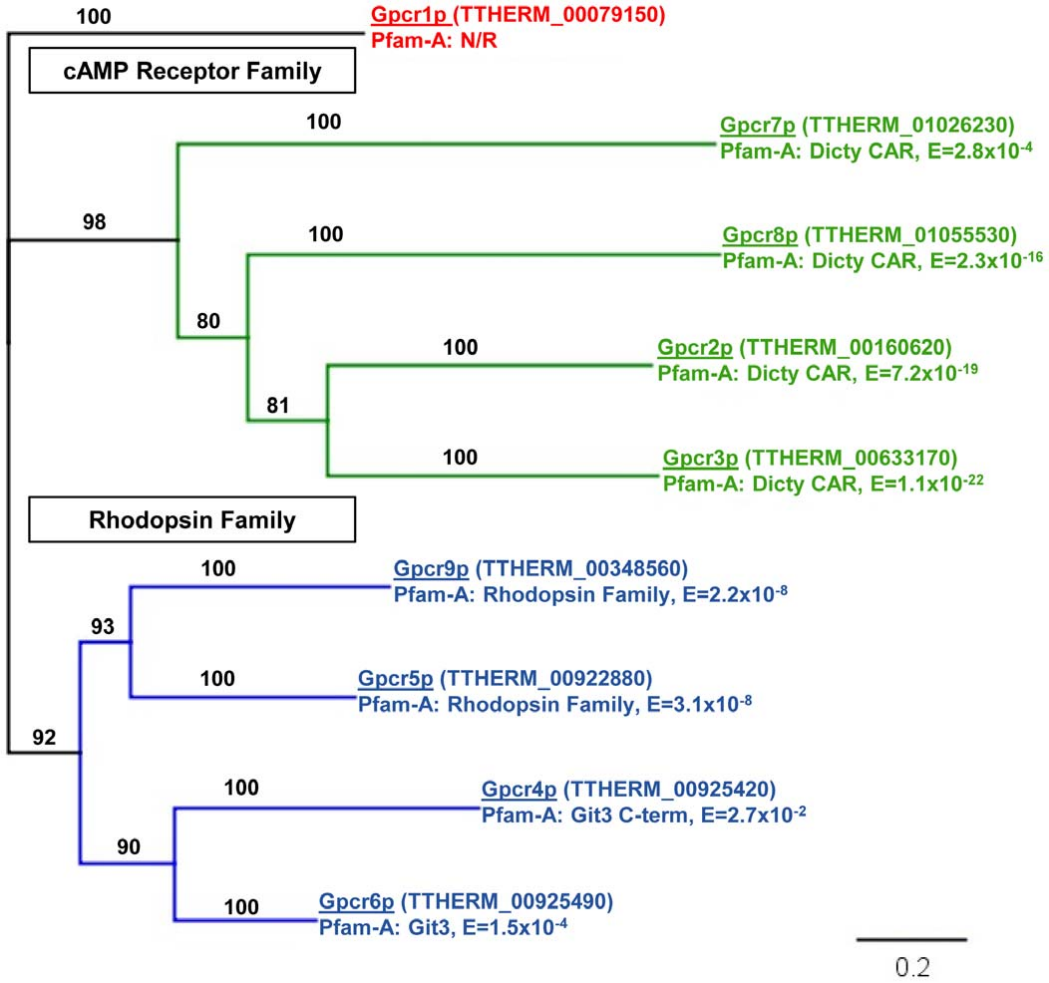
Putative *Tetrahymena* GPCR amino acid sequence analysis. Neighbor-Joining phylogenetic tree depicting the relationship of the 9 *Tetrahymena* GPCR candidates. Two major clades are evident and supported when analyzing the sequences through the PFAM database: cAMP Receptor Family (specifically the Dictyostelium CARs) and the Rhodopsin family. The Gpcr6p protein falls into the Rhodopsin Family clade with a significant domain related to fungal nutrient receptors (Git3). Scale bar represents substitutions per site.

Nine candidate GPCRs were selected from the *Tetrahymena* Genome Database (TGD, http://www.ciliate.org/). There may be more GPCRs in the *Tetrahymena* genome but this represents the strongest group of candidates. A phylogram depicting the relationship of these nine putative GPCR amino acid sequences is presented in Figure 1. The phylogram depicts two clades that are not only supported by the topology of the resulting tree but also by domain annotations from the PFAM server (http://pfam.sanger.ac.uk/). One clade shares varying homology to *Dictyostelium* cAMP (CAR) receptors [21]. The second is divided into two smaller clades, one rhodopsin-like [37] and the other related to the yeast nutrient receptors [19]. The amino acid sequence of the Gpcr6p protein (TTHERM_00925490, XP_001031166) shares significant homology with the Git3 nutrient receptor from *Schizosaccharomyces pombe* [20].

**Figure 2:**
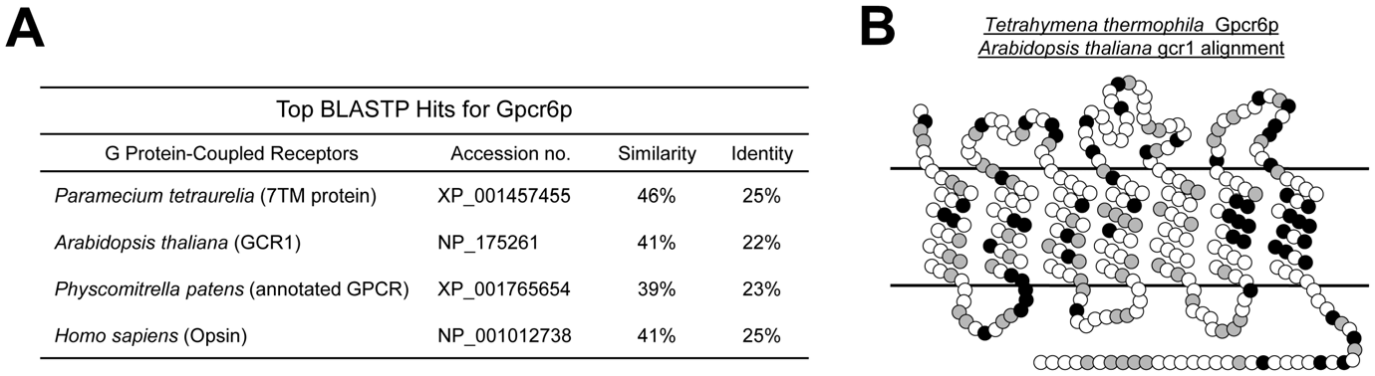
Gpcr6p homologies to sequenced genomes. **A.** The GPCR6 translated animo acid sequence shows a limited homology to other eukaryotic GPCRs. This protein may have evolved to specifically address a ciliate function from an ancestral proto-GPCR. **B.** Several algorithms were used to identify potential membrane spanning segments of the Gpcr6p protein. Based on these predictions, the membrane topology of the receptor was deduced (Figure 2B). All prediction methods arrived at a heptahelical membrane protein. The results of a ClustalW alignment between Gpcr6p and the gcr1 receptor from *Arabidopsis thaliana* is represented by the filled circles: grey are similar and black are identical amino acids from the alignment. The strongest homologies were observed in transmembrane domains VI and VII. The top side represents the extracellular side.

The top NCBI BLAST hits for the predicted Gpcr6p amino acid translation are displayed in Figure 2A. All of the BLAST results are weakly significant, even for the hit from the most closely related *Paramecium* genome. The *Arabidopsis* GPCR is the best reciprocal blast hit (BRH) outside of the Phylum Ciliophora. This result could have been predicted as there is evidence that ciliates are more related to plants than they are to animals and fungi [38]. Membrane prediction algorithms were also used to define the GPCR associated seven-transmembrane-domain topology for Gpcr6p (see methods). The consensus transmembrane prediction was used to deduce the membrane topology of the protein (Figure 2B). The black circles represent identical residues, shaded circles represent conservative substitutions of an amino acid sequence alignment between *Arabidopsis* gcr1 and *Tetrahymena* Gpcr6p. There is little overall homology except for transmembrane regions VI and VII. These transmembrane domains have been reported to be involved in the interaction and altering of G**α** activity [39]. In addition, these transmembrane domains are also the most conserved regions between all the candidate *Tetrahymena* GPCRs (Figure S2A).

The short amino terminus of the predicted Gpcr6p peptide is indicative of Class A, Rhodopsin-like Family GPCRs. The ligand binding region for this class is generally within the core of the receptor [17], [40]. Two different GPCR prediction and classification servers were utilized and they both supported placing Gpcr6p in the Rhodopsin Family [41], [42]. Moreover, the Gpcr6p amino acid sequence contains a cysteine residue in the first and the second extracellular loops. Cysteine residues in these two extracellular loops have been demonstrated to be essential in maintaining the structural integrity of GPCRs; specifically Rhodopsin Family proteins via disulfide bonds [43]. Beyond the Rhodopsin-like family GPCRs, these cysteine residues are also conserved in the fungal nutrient receptor family [20]. These cysteine residues are conserved in all the *Tetrahymena* GPCR candidates and thus provide additional support for a *Tetrahymena* GPCR family containing multiple genes (Figure S2B).

### Construction and confirmation of the GPCR6 macronuclear knockout

**Figure 3:**
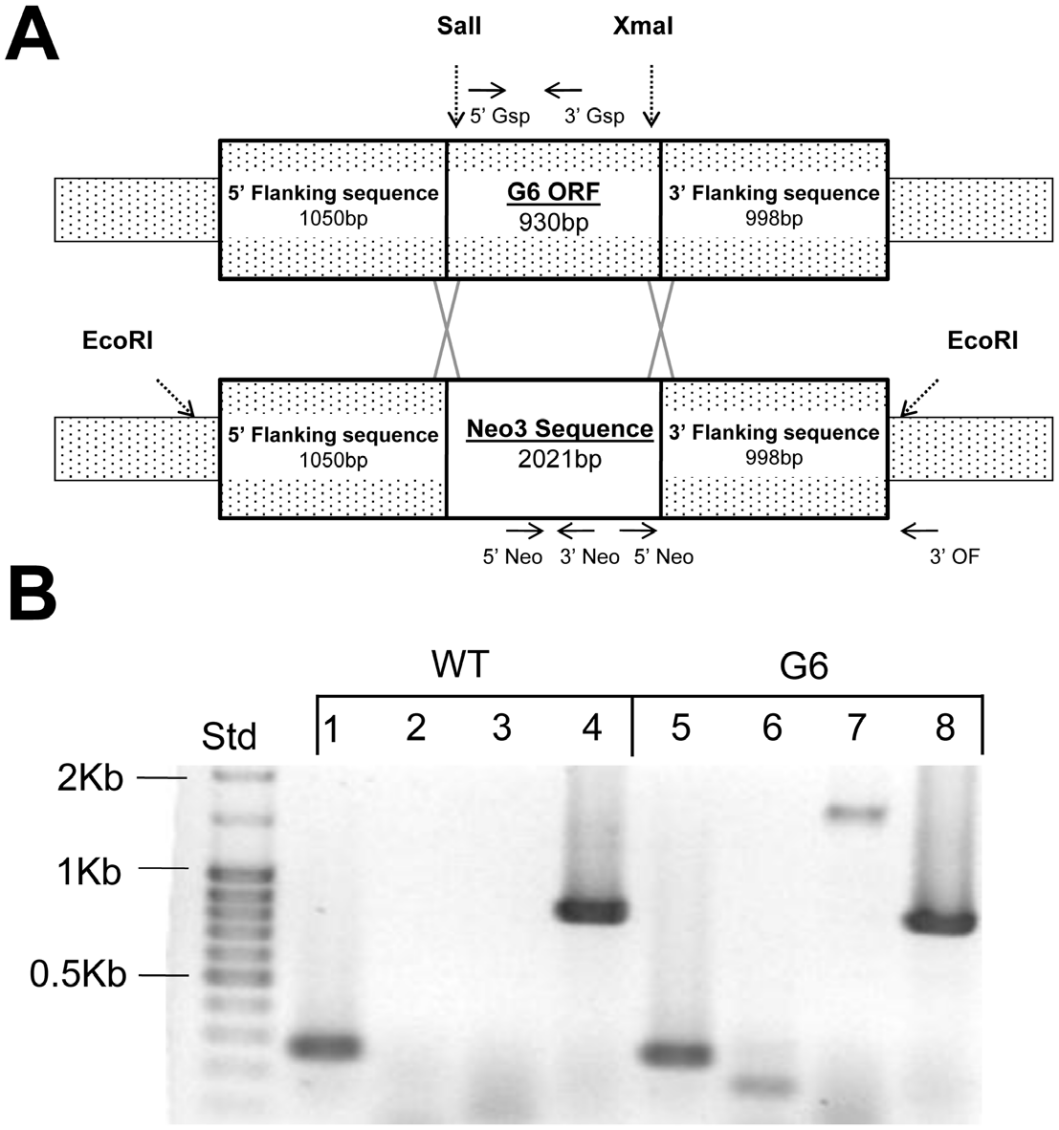
Creation of the GPCR6 knockout mutation. **A.** Diagram of the genetic construct used for homologous recombination in disrupting the GPCR6 coding sequence. The genomic coding regions of GPCR6 (TTHERM_00925490), along with about 1 kb of flanking sequences on both sides, were cloned into a TOPO vector for modifications. Restriction sites (*SalI* and *XmaI*) were added near both the 5′ and 3′ ends of the coding regions by site-directed mutagenesis. The neo3 antibiotic resistance cassette was cut from its vector with the same restriction enzymes and was ligated into the TOPO vector to replace the coding regions with the antibiotic resistance cassette. The completed GPCR6 knockout construct is shown above. The linearized knockout construct was introduced into vegetative CU427 wild-type cells by biolistic transformation. **B.** Genomic PCR was used to confirm the correct disruption of the GPCR6 coding sequence. Lanes 1–4 are PCR products from wild-type (WT) DNA and lanes 5–8 are from G6 DNA. GPCR6 gene specific primers were used in lanes 1 and 5. The wild-type product (252 bp) is seen in Lane 1. Lane 5 shows the same band because the wild-type GPCR6 sequence is still present in the micronucleus of G6 (it is a macronuclear knockout). Neo3 primers in lanes 2 and 6 shows that neo3 is present in G6 (lane 6) but not wild-type (lane 2). Lanes 3 and 7 paired a 5′ neo3 primer with a 3′ outer flanking primer showing that G6 has this 1,753 bp product (lane 7) but the wild-type doesn't (lane 3). Lanes 4 and 8 are bands generated from control primers (RPL21) for a ribosomal protein gene.

**Figure 4:**
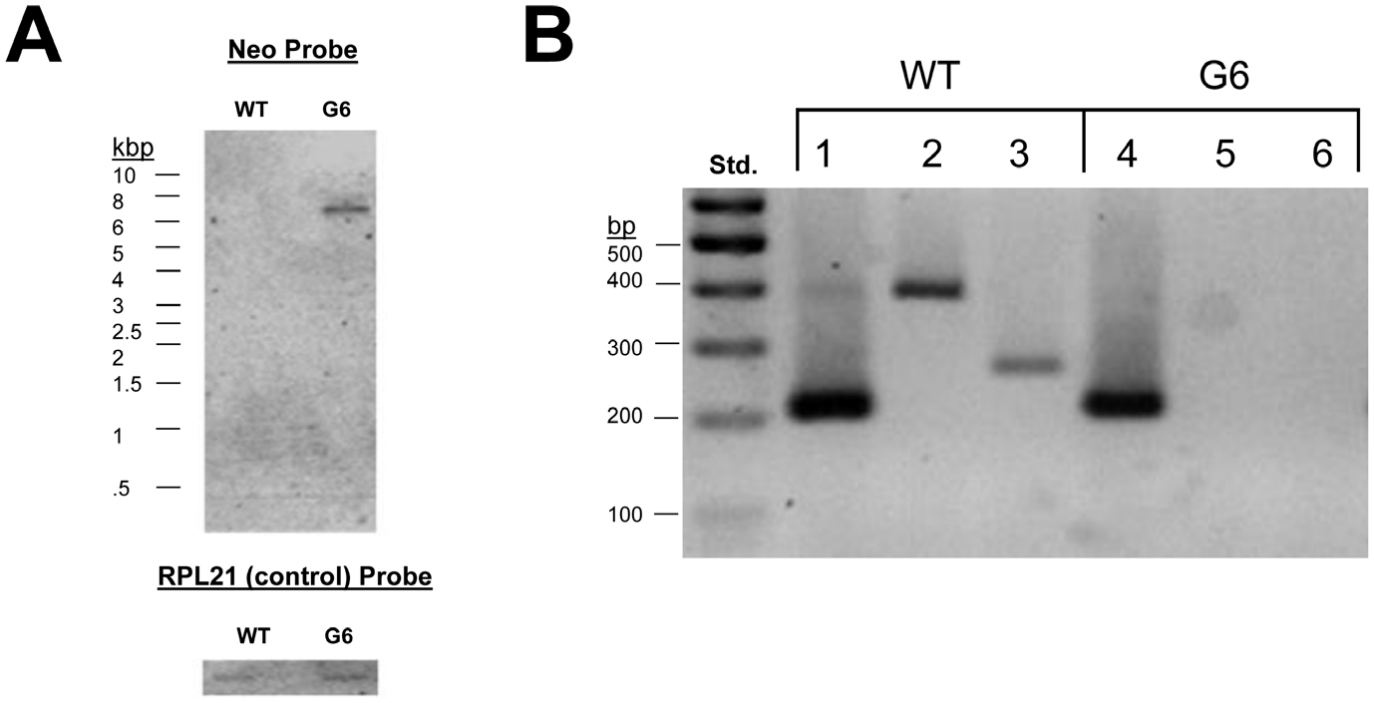
Confirmation of the G6 knockout cell line. **A.** Southern blot of genomic DNA from wild-type (WT) and G6. An epitope labeled DNA probe was made to recognize the neo3 cassette sequence with the DIG probe labeling kit by Roche (top gel). Another probe was made to recognize the ribosomal protein subunit gene RPL21 as a control (bottom image). Restriction digests were performed with *EcoRI* before agarose electrophoresis and blotting. Hybridization of the probe to a blot was visualized by exposing the blot to film. The G6 mutant had only one band at the size predicted for the neo3-containing *EcoRI* fragment (6.7 kb) showing that the mutant was generated by homologous recombination into only one gene. **B.** RT-PCR on RNA extracted from WT and G6 cell lines followed by PCR on cDNA using gene specific primers to examine gene expression. Lanes 1 and 4 are RPL21 controls. Lanes 2 and 5 are gene specific primers, gsp1 for GPCR6 while lanes 3 and 6 are a different set of Gsp's for GPCR6. Both GPCR6 specific primer sets show that GPCR6 is not expressed in G6 mutants.

Once the biolistic knockout transformation was complete, the resulting resistant cell lines were grown under increasing concentrations of paromomycin. The cell lines were kept in paromomycin until selection led to fixation of the knockout allele [35], [36], [44]. For confirmation of the G6 knockout mutant, genomic DNA was compared to wild-type (WT) using PCR. The DNA primers used are depicted in Figure 3A in relation to the knockout locale. Use of the Neo and OF (outer flanking) primers in PCR reactions on G6 derived DNA demonstrates both the presence of the neo3 sequence as well as the correct insertion in the intended genomic location (Figure 3B). The Gsp's (gene specific primers) demonstrates that the GPCR6 sequence is still present in the mutant because the DNA preparation contains both macronuclear and micronuclear (which is wild-type in G6) genomic DNA. Southern blotting using a probe designed to hybridize to the Neo ORF was used to assure that no other recombination events occurred. The neo probe hybridized to a band in the range of the 6,728 bp predicted restriction fragment of the recombined knockout locale (Figure 4A). This blot was stripped and re-probed for RPL21 (a ribosomal protein gene), a control that appeared in both WT and G6 lanes.

The extent of the knockout mutation was analyzed by RT-PCR to see if there was any expression of the GPCR6 gene in G6. The cDNA of both WT and G6 cells was subjected to PCR using specific primers to the predicted GPCR6 mRNA sequence. Figure 4B shows the results of two sets of different gene specific primers. No GPCR6 PCR products are observed for the G6 cDNA, suggesting G6 is a complete knockout of GPCR6. The RPL21 primers confirmed the correct processing and stability of the isolated mRNA because the PCR products showed the correct sizes for the predicted spliced products. G6 showed no detectable level of GPCR6 expression compared to WT from several samplings over months out of paromomycin selection, showing that the knockout was complete and stable.

### Alterations in excitability

Behavioral responses to Ba^2+^ and K^+^ have been historically used in analyzing excitability changes in behavioral mutants of *Paramecium* [45] and barium paralysis has been used to screen for possible mutants in calcium channel activity [46]. The barium paralysis assay solution is a modification of the Dryl's medium containing 10 mM Ba^2+^. When G6 was incubated in the Ba^2+^ paralysis solution, the cells were resistant as measured by changes in swim speed (Table 1), suggesting a possible defect in excitability. In high concentrations of Ba^2+^ or K^+^, cells swim backwards for several seconds. These long durations of backward swimming are due to continuous ciliary reversals (CCR). The G6 mutant shows a decreased duration of backwards swimming (CCR) in either 20 mM K^+^ or 1.0 mM Ba^2+^ (Table 1). The duration of CCR in Ba^2+^ is almost completely lost in the G6 mutant while the duration of K^+^-dependent CCR is decreased but not eliminated. These results suggest a constitutive role of GPCR6 in regulating excitability and Ca^2+^ channel activity.

**Table 1:** Physiological Screen for G6.

Condition	WT	G6
Basal Swim Speed	0.43±0.07 mm/sec.	0.44±0.08 mm/sec.
Swim Speed in Ba^2+^	0.10±0.04 mm/sec.	* 0.40±0.04 mm/sec.
Swim Speed in PP	0.64±0.09 mm/sec	0.63±0.07 mm/sec
Basal V_m_	−28.5±2.70 mV	−27.4±1.52 mV
V_m_ in Ba^2+^	−20.2±3.96 mV	−19.9±2.82 mV
AR in 0.1 mM Ba^2+^	100±10%	90±6%
AR in 2.5 mM K^+^	80±5%	87±6%
CCR in 1 mM Ba^2+^	17.5±2.00 sec.	* 3.37±3.80 sec.
CCR in 20 mM K^+^	22.2±2.60 sec.	* 12.6±1.99 sec.

*p<0.05, students t-test.

Swim speeds: n = 3 experiments ∼30 cells each.

V_m_: basal; n = 6, Ba^2+^; n = 5.

AR/CCR: n = 3 experiments, 10 cells each.

Resting membrane potential recordings demonstrated that under basal and Ba^2+^ depolarizing conditions the somatic membrane potential of G6 is similar to wild-type (Table 1), suggesting no major changes in resting conductances. This is also reflected in the observation that the swim speeds in these solutions also did not differ between G6 and wild-type (Table 1). The cell's ability to show avoiding reactions (AR) is a bioassay for their ability to generate action potentials. The percent of cells showing AR in Ba^2+^ and in K^+^ were not significantly different between the wild-type and the G6 mutant (Table 1), showing that they are capable of generating AR. Since there were no differences between G6 and wild-type AR but there were differences in their durations of CCR in higher Ba^2+^ or K^+^, it appears that the defect in G6 is not in their ability to generate action potentials but rather in their ability to sustain them.

### Gpcr6p signaling is required for chemoattraction in *Tetrahymena*

Our model is that both chemoattractants and chemorepellents have their behavioral effects by changing basal excitability, reflected in the percent of cells showing directional changes (PDC). If chemoattractants and chemorepellents are to have opposite effects on the PDC of *Tetrahymena*, then the basal excitatory behavioral state should be maintained between these two states. In this manner, chemoattractants can decrease basal PDC to bias the swimming paths to be straighter towards the attractant while repellents increase PDC to send these cells away from noxious stimuli [1], [6], [47]. We propose that Gpcr6p is required for a constitutive signal that is integral in maintaining proper basal swimming behavior in *Tetrahymena*.

**Figure 5:**
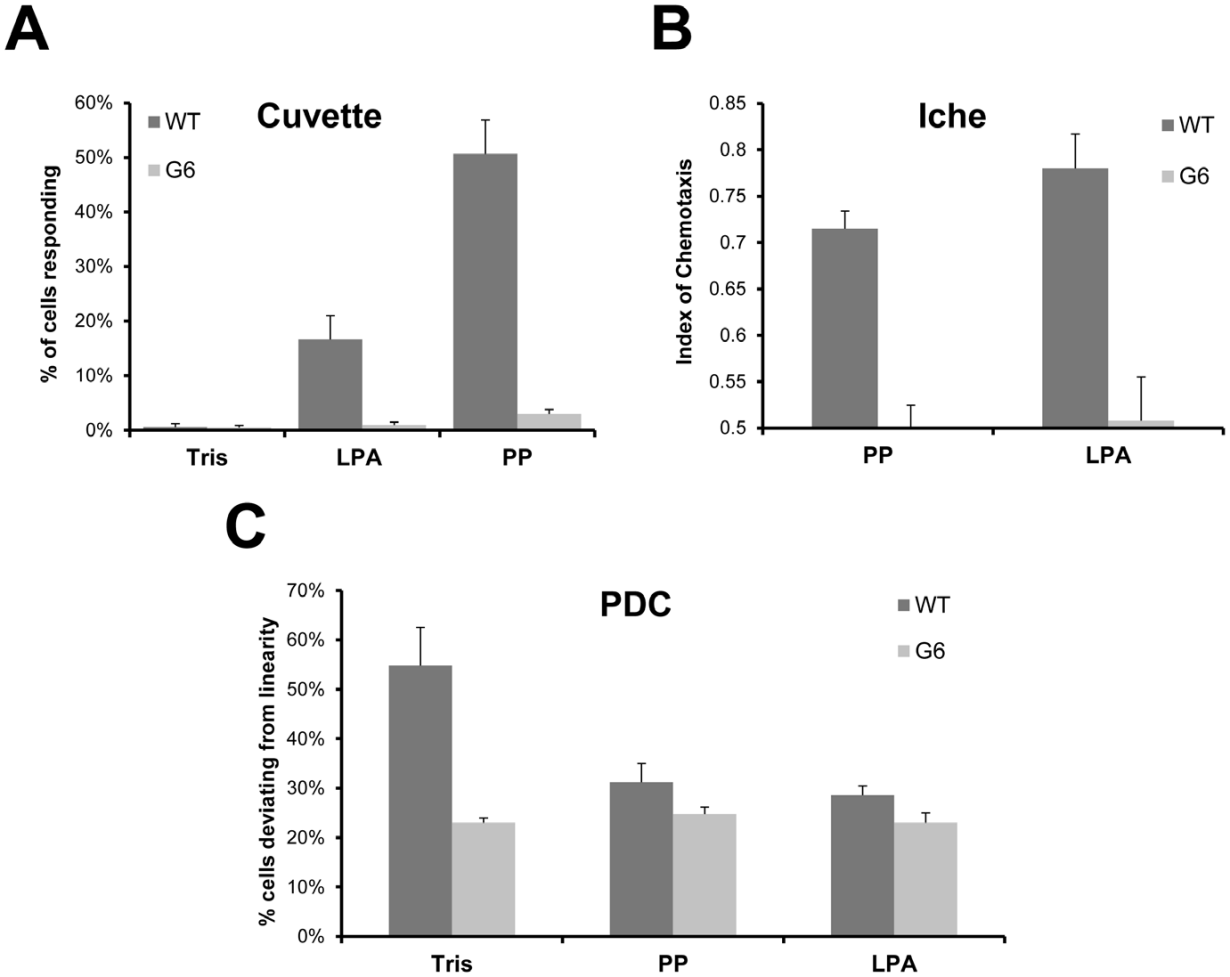
Chemoattraction behavior is altered in the G6 knockout cell line. **A.** The chemoattraction assay commonly used in *Tetrahymena* (two-phase assay) showed that proteose peptone (PP, 1 mg/ml) and lysophosphatidic acid (LPA, 10 µM) are chemoattractants for wild-type two-day starved cells. Data represents the % of cells that accumulated in the lower phase after 30 min., n = 3 separate cultures. Chemoattraction responses were not present in the G6 mutant cell line. **B.** Three-way stop cock assay typically used for ciliate behavior analysis. While wild-type showed chemoattraction to either 1 mg/ml PP or 10 µM LPA, G6 is significantly less than wild-type in both cases. The G6 mutant showed no attraction towards either PP or LPA, n = 5. **C.** Transfer of cells from the control solution (Tris) to either PP or LPA significantly decreases the percent of directional changes (PDC) in wild-type. A decrease in PDC has been associated with chemoattraction responses in ciliates. The G6 mutant does not show a significant decrease in PDC because the PDC level is already low. n = 3 experiments, ∼30cells each. Significance determined by student t-test<0.05 with bonferroni correction where applicable.

**Figure 6:**
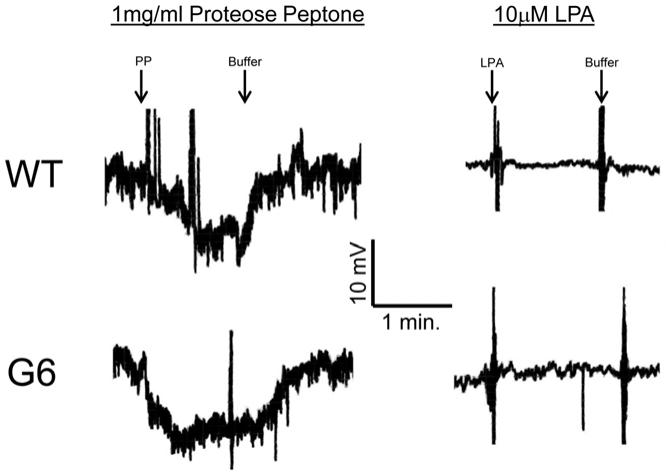
The electrophysiological responses to proteose peptone (PP) and lysophosphatidic acid (LPA) of G6. PP causes a large and reversible hyperpolarization in both wild-type and in the G6 mutant. However, the G6 mutant cannot show chemoattraction to PP. LPA does not cause any changes in membrane potential in both wild-type and G6 cells. The large upward and downward spikes are perfusion artifacts that were unavoidable during changes in bathing solutions. Figure representative of three similar traces.

The G6 mutants showed no measurable chemoattraction (compared to wild-type) to either lysophosphatidic acid (LPA) or proteose peptone (PP) in three different behavioral bioassays. The first is a two-phase spectrophotometric assay that shows chemoattraction as an increase in A_600_ as cells move past the light path in a cuvette [5], [8] (Figure 5A). The second chemoattraction assay is the three-way stop cock assay used in *Paramecium* [47] (Figure 5B). The G6 cells' basal mobility is the same as in wild type in both of these chemoattraction assays (Figure S3). The third is an assay that measures the percent of cells showing direction changes (PDC) [6], [9] (Figure 5C). Although all three rely on different criteria for chemoattraction, they all show that wild-type cells respond well to the attractants LPA and PP but G6 cells do not. The G6 mutant could change their swim speed in response to stimuli. In response to PP, the membrane potential hyperpolarized in the G6 mutant (Figure 6) and their swim speed increased from 0.44 mm/sec. to 0.63 mm/sec. (Table 1). These results are the same as the wild-type responses to these chemoattractants. Even though the G6 mutants were able to hyperpolarize and increase their swim speeds in response to PP, they still did not show an attractant response, suggesting that swim speed changes are not always necessary for chemoattraction in *Tetrahymena* [6], [48].

### Changes in the G-protein activity in microsomes

**Figure 7:**
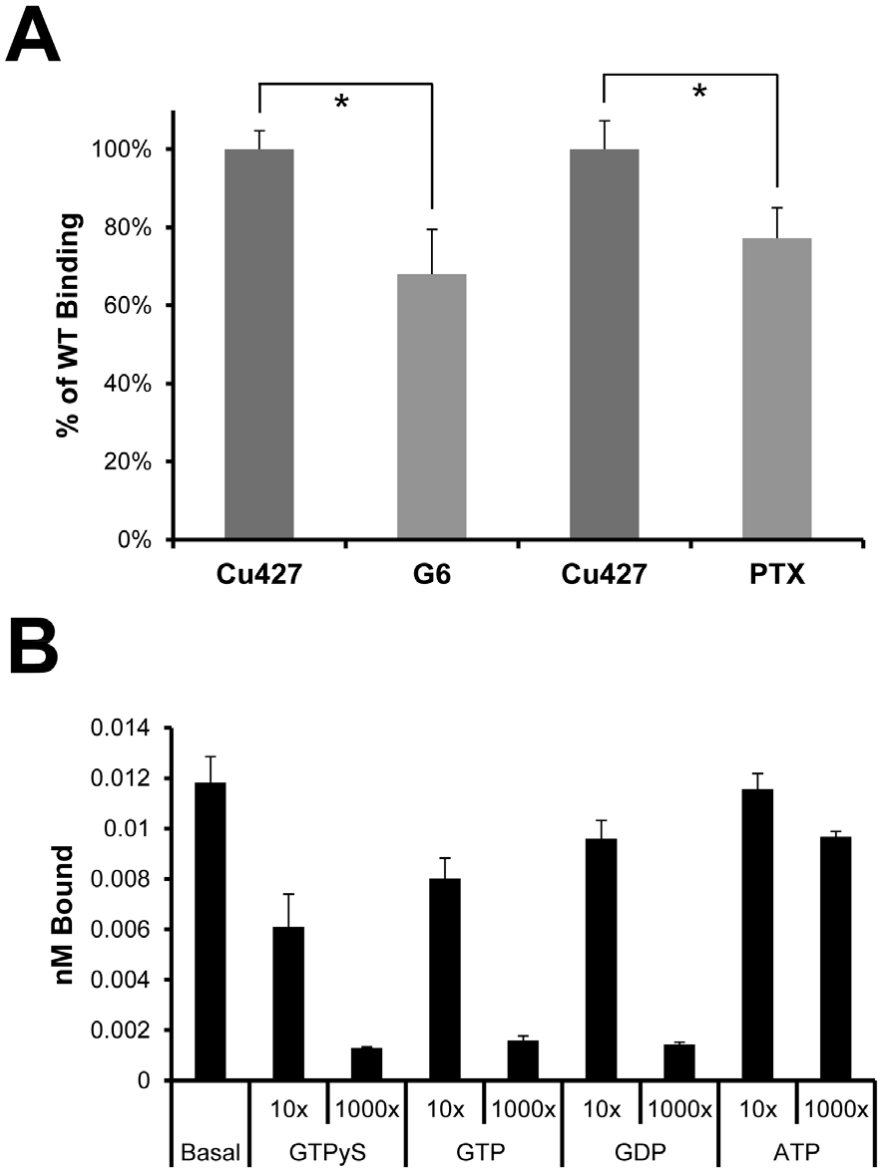
Analysis of G-protein activity in *Tetrahymena* microsomes. **A.** 0.1 nM [^35^S]GTP**γ**S binding to G6 microsomes shows a significant decrease in G-protein activity compared to wild-type. Microsomes from PTX treated cells showed a similar decrease in G-protein activity. This suggests that both conditions affect GPCR signaling by decreasing the coupled G-protein activity. n = 3 determinations of 3 different membrane preparations (* p<0.05, t-test). **B.** While GTP**γ**S, GTP and GDP competed well for [^35^S]GTP**γ**S binding, ATP shows little competition and therefore strengthens the support for specific GTP binding proteins. ANOVA and Dunnet's test (95 and 99% C.I.'s) were used to determine significance compared to control.

[^35^S] GTP**γ**S binding to isolated cell membranes is an established method for measuring G-protein activity from both cells and tissues preparations, specifically in reference to GPCR signaling [49]. *Tetrahymena* microsomes from two-day starved cells were prepared in order to examine G-protein activity in relation to reported behavior. Microsomes isolated from G6 and wild-type cells treated with PTX both displayed a significant decrease in GTP**γ**S binding compared to wild-type control (Figure 7A). Pertussis toxin causes ADP-ribosylation of G-proteins and thus prevents the interaction with GPCRs [50]. Addition of the chemoattractant LPA to the microsome binding reactions also decreased the basal G-protein activity to a similar extent. LPA added to G6 microsomes, LPA added to PTX treated wild-type cell microsomes, and PTX treated G6 cells all showed no significant difference in the level of decreased G-protein activity (ANOVA): %WT control = 65±5%, 64±8%, 65±5%, 64±3% for LPA, G6+LPA, PTX+LPA, and G6+PTX respectively (n = 3 microsome preparations). These results suggest that all three of these conditions affect the same PTX-sensitive G-protein activity. The addition of 1 mg/ml proteose peptone further decreased the GTP**γ**S binding in wild-type (data not shown). Due to the complex composition of this proteose peptone solution, we did not pursue these results further. Figure 7B provides support for the specificity of the proposed G-protein binding of GTP**γ**S to membranes by competition.

**Figure 8:**
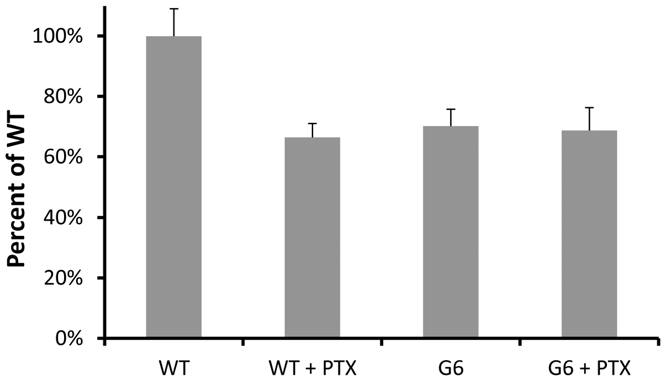
Activated pertussis toxin treatment on the G-protein activity of microsomes. As an alternative to treating whole cells with PTX, *Tetrahymena* microsomes were treated with activated toxin. As before, G6 microsomes showed decreased binding but there was no decrease when treated with activated PTX. This suggests that the Gpcr6p pathway is working exclusively through a PTX sensitive G-protein. (n = 3 determinations for 3 membrane preparations). Significance determined by ANOVA, Tukey multiple pair wise analysis (95% and 99% C.I.'s).

Treatment of microsomes with activated PTX also provided additional support for these conclusions. The activated toxin can be used to treat duplicate samples of the same membrane preparation to remove any variability between microsome isolations. Treatment of wild-type membranes with activated PTX decreases the G-protein activity to the same extent as when whole living cells were treated with the toxin (compare Figure 7A and Figure 8). Unlike the wild-type, the G-protein activity in the G6 microsomes was not significantly decreased by activated PTX treatment (Figure 8). This provides further support that the basal, constitutive activity of wild-type Gpcr6p most likely engages PTX-sensitive G-proteins.

## Discussion

### Evidence supporting GPCR6 placement in the GPCR Superfamily

Since members of the GPCR Superfamily are utilized in chemosensory transduction for many types of eukaryotic cells [15], [18], we screened for possible GPCRs in the *Tetrahymena* Genome [51]. Although the known members of the GPCR Superfamily all possess a seven transmembrane topology, an apparent seven transmembrane domain structure does not necessarily indicate heterotrimeric G-protein signaling. There are many studied “GPCRs” that have been reported to initiate a wide range of intracellular signaling events through G-protein-independent effector molecules [52].

Hydropathy analysis and amino acid alignments to the transmembrane domain of known GPCRs provides strong support for *Tetrahymena* GPCR6 encoding a heptahelical membrane protein. The reciprocal BLAST results between Gpcr6p of *Tetrahymena* and current sequences in other databases showed that Gpcr6p has similarities to *Paramecium* and to possible GPCRs in plants (Figure 2A). Similarities to gcr1 of *Arabidopsis* suggests that this gene could be part of the family of receptors sharing a most recent common ancestor prior to the divergence of Archaeplastida and Chromoalveolates [53].

GPCR pathways have been well studied in plants but the ligands and most of the downstream signals have yet to be fully elucidated [33], [54]. The putative *Arabidopsis* GPCR called GCR1 interacts with prototypical G-proteins and gene disruption experiments induced phenotypic defects in development and transpiration [33], [55]. The transpirational aspects have been linked to cation channel regulation by G-protein signaling in plants [56]. This suggests a possible role for Ca^2+^ regulation by GPCRs.

Two cellular functions that are essential to many protozoan species are the acquisition of nutrients and sexual reproduction. Not surprisingly, these are essentially the two GPCR pathways that have been elucidated in *Saccharomyces* species. The PFAM annotations of the two main clades in the *Tetrahymena* GPCR phylogram appear to suggest homologies to the Git3 nutrient receptors of yeast and the cAMP receptors required for sexual development in *Dictyostelium* (Figure 1). All of the genes in the *Tetrahymena* cAMP clade show substantial changes in expression during mating while the expression of GPCR6 is relatively unchanged during growth, starvation, and mating [57]. G6 cells also showed normal conjugation and mating behaviors, thus further separating the G6 pathway from the mating signaling events (data not shown). But, as the knockout mutation is in the macronucleus there may be GPCR6 dependent events further into sexual reproduction that would only be observed with a micronuclear knockout.

Based on the domain similarity with the fungal nutrient receptors, one hypothesis would be that Gpcr6p is directly responsible for detection of carbohydrates, amino acids, lysolipids, or other nutrients [19], [20], [58], [59]. The nutrient GPCRs in yeast may detect a variety of nutrients, with low affinities for each, allowing for broad range nutrient detection [19]. This type of broad nutrient reception receptor system might also be advantageous to *Tetrahymena*. The results of this study do provide evidence that suggests a mechanism in which Gpcr6p is a nutrient receptor. Chemoattractants like the amino acids in proteose peptone or LPA may act as inverse agonists for constitutive G-protein activation by Gpcr6p. Cell motility alteration and chemoattraction occurring through inverse agonist effects on constitutive GPCR signaling has been previously reported in cultured vertebrate cells [31], [32]. LPA is known to be an attractant for many cells that are prokaryotic predators [59], [60]. Therefore, LPA could represent a reasonable nutrient chemoattraction signal for *Tetrahymena*. Binding of ^3^H-LPA to the G6 mutant cells was examined, but the lipophilic nature of the compound produced high nonspecific binding to the samples. Therefore, at present it is not possible to decided unequivocally whether Gpcr6p is the LPA receptor or if LPA modulates the basal activity of G-proteins downstream of the receptor [61].

### G-protein activity and behavioral responses altered by GPCR6 KO

The importance of endogenous inverse agonists and antagonists in GPCR signaling is only recently being truly appreciated. The discoveries that constitutive GPCR signaling is much more prevalent than previously predicted and the description of the effects of inverse agonists have changed the classical view of GPCR functions [30], [62]. The constitutive nature of Gpcr6p is seen by comparing wild-type with the G6 mutant because the knockout phenotypes are seen without the addition of known ligands. Gpcr6p also has the lowest level of relative expression compared to the other eight candidate GPCRs [57]. A low level of basal expression is indicative of many constitutive GPCRs [17], [63]. The prototypical plant G-protein possesses intrinsic constitutive activity and the specifics of its signaling mechanisms are still unknown [64]. As this G-protein is known to interact with a putative plant GPCR this may represent a novel paradigm of how a receptor will regulate the activity of a G-protein [33].

**Figure 9:**
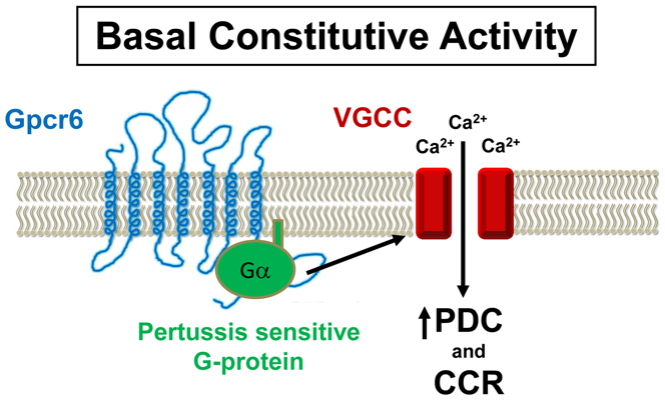
Proposed signaling model. The wild-type Gpcr6p causes constitutive activation of a pertussis sensitive G-protein that modulates the opening of the ciliary voltage-gated Ca^2+^ channels (VGCC). Since these channels provide the Ca^2+^ for Ca^2+^-dependent ciliary reversals, this makes the basal percent of cells showing direction changes (PDC) relatively high in unstimulated wild-type. Chemoattractants act as inverse agonists to decrease G-protein activation, lower the basal PDC and cause straighter forward swimming towards the attractant. Strong depolarizations cause prolonged Ca^2+^ channel activation, continuous ciliary reversals (CCR) and backward swimming. In both the G6 mutant and the wild-type with pertussis toxin, this G-protein activation is missing so there is a lower basal PDC, no chemoattraction and decreased CCRs in high concentrations of either Ba^2+^ or K^+^.

In our model (Figure 9) wild-type Gpcr6p affects the basal voltage-dependent Ca^2+^ channel activity through the activation of a PTX-sensitive G-protein. We suggest that this could be a G**α** because there are some possible candidates in the database but no G-protein has been molecularly identified yet in *Tetrahymena*. This model is supported by the observations that the G6 mutant, which does not express Gpcr6p, has a decreased basal G-protein activity (Figures 7,8), lower basal PDC (Figure 5C), loss of chemoattraction (Figures 5A,B) and decreased responses to Ba^2+^ and K^+^ (Table 1). These same effects can be seen in wild-type with PTX (Figures 5,7,8) [6], suggesting that the G6 mutant is lacking the PTX-sensitive component of the wild-type basal G-protein activity. The behavioral responses of G6 (Figure 5) suggest an effect on the voltage-dependent Ca^2+^ channels [65] because these channels are required for generating ciliary reversals [45]. Presumably, this also happens in *Tetrahymena* because a mutant that lacks Ca^2+^ action potentials also has defective responses to Ba^2+^ [66]. Consistent with this, it was shown in *Paramecium* [27] that G-protein activation increased the amplitude of the inward Ca^2+^ current without changing the voltage sensitivity. Since Ba^2+^ or K^+^ can still produce reliable responses in the G6 mutant (Table 1), they are obviously capable of Ca^2+^ channel activation. However, their low basal PDC suggests a reduced Ca^2+^ activity and decreased excitability. There are also suggestions that some GPCRs may themselves be ion channels [67], [68] and yet they may function through both direct ion conductance and heterotrimeric G-protein regulation [69], [70]. Since neither intracellular voltage clamp or patch clamp procedures are well described in *Tetrahymena*, further detailed analysis of Ca^2+^ channel properties in intact cells is not currently feasible.

The [^35^S]-GTP**γ**S binding of microsomes was decreased reliably by either addition of PTX to wild-type or by the G6 mutation. To control for possible variability between microsome preparations, each of the [^35^S]-GTP**γ**S binding assays were done with the same microsome preparation except that activated PTX was added to one half. This way, the effects of PTX could be directly evaluated in identical microsome samples. This was repeated with three different microsome preparations and summarized in Figure 8, showing that PTX causes a reliable decrease in [^35^S]-GTP**γ**S binding in wild-type but not in the G6 mutant. There was also no difference between wild-type with PTX and G6. All together, this suggests that it is the PTX sensitive component of wild-type that is missing in Gpcr6p.

For Gpcr6p to be defined as a prototypical GPCR, ligand-induced changes in G-protein activity must be shown. To address possible G-protein activity, [^35^S]GTP**γ**S binding assays were performed on *Tetrahymena* microsomes. These experiments showed that Gpcr6p signals through a PTX sensitive G-protein pathway (Figures 7,8). Moreover, it appears to be the same G-protein component that is decreased when the chemoattractant LPA is present. These results can explain the behavioral results of the G6 and PTX treated wild-type cells (Figure 5) (Table 1) [6] as follows. Wild-type have a constitutive Gpcr6p activated, PTX-sensitive G-protein activity that is decreased by the chemoattractants LPA and PP. This causes decreased Ca^2+^ channel activation, fewer ciliary calcium fluxes and less random directional turns. This results in longer bouts of straight-forward swimming towards an attractant. This concept is similar to the tumble and run model for bacterial chemotaxis [71] because both involve changes in the frequency of direction changes.

Although *Tetrahymena* have the advantages of free-living unicellular simplicity, high cell yields from axenic cultures, gene knockouts and a strong history of biochemical, molecular and behavioral characterizations they also have a disadvantage of difficulties in heterologous expressions. Because they have a different genetic code [72], heterologous expression requires extensive site-directed mutagenesis. In addition, differences in RNA and protein processing, protein trafficking, and other cellular differences create additional problems for functional heterologous expression of *Tetrahymena* gene products. Since very few *Tetrahymena* membrane proteins have been shown to be heterologously expressed, properly localized and functional, this approach is currently problematic. However, since the *Tetrahymena* genome contains at least eight additional putative GPCRs, several possible G-proteins and many other likely effectors, this current study can provide the basis for future research that could produce insights into both the functions of GPCR pathways in *Tetrahymena* and the evolution of the GPCR Superfamily in eukaryotes.

## Materials and Methods

### Cell stocks, culture, and maintenance

*Tetrahymena thermophila*, stock CU427 (Cornell University *Tetrahymena* Stock Center) were used for wild-type controls and creation of the biolistic transformed knockout mutants. All cells were cultured in 5 ml SPP media (1% proteose peptone, 0.2% glucose, 0.1% yeast extract) in standing tubes at 25°C. Transformed cells were maintained in SPP supplemented with 100 ng/ml paromomycin and 0.5 µg/ml CdCl_2_. Mutant cell lines were kept under increasing paromomycin selection until expression of the targeted gene was no longer observed. Several of the assays required the cells to be washed and starved for varying periods of time in assay buffer (10 mM Tris, 50 µM CaCl_2_, pH 7.2 with MOPS). For the 5 ml cultures, cells were washed in 100 ml sterile assay buffer at 500 g. The cell pellet was removed and left in this buffer for at least 30 min. before using in assay. Other assays required 50 ml cultures which were washed in the same way except the pellet was then placed back into a sterile flask containing 50 ml of the assay buffer. These cells were then placed aside to starve for a period of time. Cells for chemoattraction experiments were starved for 40 hours at 25°C without agitation.

### Candidate gene selection and in silico analysis

The candidate GPCR genes were selected based on initial annotation of the *Tetrahymena* genome database (http://www.ciliate.org/) [51] and BLAST searches into and out of the *Tetrahymena* genome. A total of 9 GPCR candidates were deduced by BLAST searches into the TGD and genome database annotations. The *Tetrahymena* GPCRs were aligned using ClustalW [73]. The consensus tree was constructed using neighbor joining and a jukes-cantor genetic distance model. Bootstrap analysis with 1,000 replications and a heuristic search method was used to create and test the topology of the phylogram in Figure 1. Protein alignments and phylogram construction was performed in the geneious bioinformatics software [74]. BLAST searches into sequenced genomes were also performed to look for distant homologies (http://blast.ncbi.nlm.nih.gov/Blast.cgi). The translated amino acid sequence of Gpcr6p was then used to predict possible structure and function. The consensus seven membrane spanning regions of Gpcr6p were derived from several available tools: TMPRED [75], Top-Pred II [76], TMHMM 2.0 [77], TM Finder [78].

### Targeted gene disruption and biolistic transformations

The sequence information from the *Tetrahymena* Genome Database was used to identify and amplify the GPCR6 genomic location by PCR (primers Figure S1). This database was used to select not only the coding regions of the targeted genes but the flanking intragenic nucleotide sequences used for homologous recombination. PCR products were then cloned into the pCR4-TOPO vector (Invitrogen). The QuickChange site-directed mutagenesis kit (Stratagene) was used to introduce *SalI* and an *XmaI* restriction sites into the cloned fragment. The neo3 antibiotic resistance cassette (gift from Martin Gorovsky, University of Rochester, NY) was cut from its vector with these introduced restriction sites and ligated into the pCR4-TOPO vector (Invitrogen). This replaced the GPCR6 coding region with the cassette which contains a *Tetrahymena* metallothionein promoter, neomycin resistance, and a BTU stop site [44]. This insertion replaced the GPCR6 coding region with the antibiotic resistance cassette. This knockout construct was introduced into vegetative CU427 wild-type cells by biolistic transformation [79].

### Knockout Genotype

To analyze and compare genotypes, the mutant and wild-type DNA was extracted using a DNA-easy kit (Invitrogen). The DNA was analyzed using PCR with primers to both the neo3 region and to the flanking region of the recombined construct. DNA sequencing of isolated PCR bands were also used to confirm the correct mutations in the GPCR6 knockout locale. To assure that the neo-construct recombined into the correct region, and only one genomic location, Southern blotting was used with a probe to the neo3 sequence. The procedure was performed as described by the DIG High Prime DNA and Detection Kit (Roche). 10 µg of genomic DNA was digested with *EcoRI* overnight at 37°C. The restriction digested DNA was then ran on a 0.8% agarose gel and transferred to a nylon membrane for Southern blotting. A 747 bp DIG labeled NEO probe was produced by the provided protocol. The probe solution was added to the prehybridization buffer treated membrane and incubated at 42°C with agitation overnight. Immunological detection with CSPD substrate was performed the next day. When imaging was complete, membrane blots were stripped with stripping buffer (0.2 M NaOH, 0.1% SDS) for 15 min. at 37°C then examined with 839 bp RPL21 probe. The expression of GPCR6 was determined by RT-PCR on cDNA templates. Total RNA from all cells types was isolated using Trizol Reagent (Invitrogen) and treated with DNase (Fermentas). RT-PCR was performed with oligo-dT primers provided with the Affinity Script kit (Stratagene).

### Electrophysiology

Standard single electrode intracellular electrophysiological analysis was used to measure whole cell membrane resting membrane potentials under perfusion conditions [13] . Cells were viewed through an inverted microscope and impaled with a single microelectrode filled with 500 mM KCl. The assay buffer was used to flood the recording bath once a cell was impaled by the electrode. For barium depolarizing conditions, assay buffer with 0.5 mM Ba^2+^ was released into the perfusion chamber. For chemoattractant responses perfusion solution contained 1 mg/ml PP or 10 µM LPA. The perfusion rate was 3 ml/min through a chamber volume of 1 ml.

### Behavioral Assays

For single cell behavioral bioassays, cells were grown in axenic culture for 40–48 hours then washed in assay buffer by centrifugation. The washed cells were given a period of 30 min. to equilibrate to the new control buffer. All tests solutions were also prepared in this assay buffer. A small fraction of the equilibrated cells were then transferred to a well slide containing 0.5 ml of the control buffer. The equilibrated cells were then transferred individually to a well containing the test solution with a pulled Pasteur pipette. The behavioral responses were then observed using a dissection microscope.

For the AR (avoidance response) assay, individual cells were scored for either showing an AR or not (+ or −) when placed in a new test solution. Avoidance responses were defined as any significant deviation from forward swimming such as brief reversals, backwards swimming, or whirling. The results are expressed as a percentage of the sample of cells displaying any of these avoidance reactions (%AR). The CCR (continuous ciliary reversal) assay employs similar methods except the results are quantified in seconds of backwards swimming. For these experiments, backwards swimming is defined as the period of time moving backwards, not the time needed to regain forward swimming.

*Tetrahymena* swimming behavior was monitored using a Moticam480 imaging system with a Boreal binocular research microscope. A 50 µl drop of cells was placed on a slide with wax circles with an inner circumference of 13 mm. The solution was spread out to assure complete contact with the inner ring and thus uniform depth between samples. Videos were taken at 40× magnification in avi-format. Forward swim speed was measured using ImageJ image processing software (http://rsb.info.nih.gov/ij/) by analyzing the lengths of the cell paths per second. The same procedure was used to assay percent directional changes (PDC). This PDC assay was a modified version of the automated cell path analysis previously described for *Paramecium* [9]. This manual quantification measured the percent of cells deviating from their linear path with a change in linearity greater than 17 degrees. The ImageJ software possesses an angle measuring tool that was used in assessing PDC.

### Chemoattraction Assays

Chemoattraction was assayed using two different experimental procedures. The first was the two-phase assay and it was performed as previously described [8]. Briefly, test compounds were dissolved in assay buffer with 3% histodenz (SIGMA) to a total volume of 1.5 ml. This was added to a cuvette and it was placed into the spectrophotometer and zeroed. The cuvette was removed and 1.0 ml of two-day-old starved cells (in the same assay buffer) was gently placed on top of the denser test solution with a Pasteur pipette. The cuvette was then placed back into the spectrophotometer and the A_600_ was used to monitor the cell response over 30 min. The results are expressed as a relative percentage of the A_600_ of the cells alone before addition to the assay.

The second chemoattraction assay was the three-way stopcock assay commonly used with *Paramecium* [47] and recently adapted for use with *Tetrahymena* [6]. Briefly, one arm of the stopcock is filled with assay buffer and another arm is filled with test solution in the assay buffer. Two-day-starved cells are placed into the third arm of the stopcock. To start the assay, the stopcock is opened and then closed after 30 min. Cells are removed from the test and control arm and counted in Lugol's stain (2% KCl, 1% iodine). The index of chemotaxis is the number of the cells in the test arm divided by the cells in the control arm plus cells in the test arm. An index >0.5 indicates chemoattraction while an index <0.5 indicates chemorepulsion.

### [^35^S]GTPγS G-protein Activity Assay

[^35^S]GTP**γ**S binding to cell membranes was performed essentially as previously described in *Dictyostelium* [80]. Pertussis toxin (PTX)(TOCRIS) treatment of whole cell was performed for 5 hrs with 100 ng/ml of the toxin. *Tetrahymena* microsomes were generated from two-day starved cells (conditions needed for optimal chemoattraction) by differential centrifugation. In brief, a cell pellet from a 50 ml culture was resuspended in 10 ml buffer B (40 mM HEPES, 0.5 mM EDTA, 250 mM Sucrose, pH 7.2 NaOH) and homogenized with a dounce homogenizer for ∼75 strokes. The homogenate was centrifuged at 17,000 g for 5 min. and then the supernatant was centrifuged at 100,000 g for 30 min. The microsome pellet was resuspended in 0.75 ml buffer B and stored in aliquots at −70°C. The BCA protein assay kit was used to quantitate the protein content in microsomes (Pierce). Each [^35^S]GTP**γ**S binding assay experiment contained 5 µg/ml membrane protein. Microsome samples were centrifuged at 10,000 g for 4 min. and then resuspended in 80 µl buffer A (10 mM KH_2_PO4, 10 mM Na_2_HPO4, 10 mM MgCl_2_) and allowed to equilibrate for 10 min. on ice before the assay. The microsomes were then incubated for 30 min. with appropriate amount of [^35^S]GTP**γ**S on ice. The non-specific binding was determined in the presence of 1 mM cold GTP**γ**S. Labeled microsomes were then collected by 10,000 g centrifugation and resuspended in 100 µl 1 mM acetic acid for scintillation counting.

As an alternative to treating whole cells with PTX, *Tetrahymena* microsomes were treated with activated pertussis toxin. Activated pertussis toxin was prepared as previously described [50]. First, 20 µg/ml of toxin was added to activation buffer (0.5 M HEPES, 0.1 M DTT, 10 mg/ml BSA, 10 mM ATP) and incubated at 30°C for 30 min. Microsomes were treated for 60 min. with 3.33 µg/ml activated toxin in ADP-ribosylation buffer (0.5 M HEPES, 0.1 M DTT, 0.1 M EDTA, 0.2 thymidine, 100 µM NAD) at 30°C. Treated microsomes were then washed with Buffer A, centrifuged at 10,000 g for 4 min. and then assayed for [^35^S]GTP**γ**S binding.

## Supporting Information

Figure S1 **Primers used in GPCR6 Knockout construction and confirmation.** Primers used for cloning and the diagnostic assays on genomic DNA and RT-PCR, cDNA templates.
(DOC)

Figure S2 **Conserved domains and residues in** ***Tetrahymena*** **GPCRs.** A. ClustalW alignment between all 9 *Tetrahymena* GPCRs for transmembrane domains VI/VII. The *Tetrahymena* Gpcr6p and *Arabidopsis* gcr1 homologous transmembrane regions VI and VII share strong homology across all *Tetrahymena* GPCRs. B. A common GPCR conserved cysteine residue is seen in extracellular loops I and II in all predicted *Tetrahymena* GPCRs. A star depicts completely conserved residues (100% similar), double dot signifies high similarity (80–100% similar), whereas a single dot represents partial conservation (60–80% similar).
(DOC)

Figure S3 **Chemoattraction control experiments.** A. By placing cells in the cuvette alone (no attractant) the rate at which they rise to the top of the solution can be reflected by a decrease in absorbance. Both wild-type (WT) and G6 cell types have the same rate of negative geotaxis (n = 3). B. The index of motility, the number of cells that moves into the experimental region of the three-way stop cock assay, is the same in the G6 mutant.
(DOC)
