# Supplementary material for: A Knockout Mutation of a Constitutive GPCR in Tetrahymena Decreases Both G-Protein Activity and Chemoattraction
Source: PLoS One. 2011 Nov 29;6(11):e28022. doi: 10.1371/journal.pone.0028022 (PMC3226668; doi:10.1371/journal.pone.0028022)
Supplement: Figure S1 — Primers used in GPCR6 Knockout construction and confirmation. Primers used for cloning and the diagnostic assays on genomic DNA and RT-PCR, cDNA templates. (DOC) [file pone.0028022.s001.doc]

| Primer | Sequence | Target | Used for: |
| --- | --- | --- | --- |
| GPCR6 5’ | 5-CAAAAAGACTACTTCAACTCA-3 | Genomic | Cloning |
| GPCR6 3’ | 5-GACGCTTCATAAAAGTACTC-3 | Genomic | Cloning |
| G6 GSP 5` | 5-TGCACATCAACTGGAATTTTG-3 | Genomic | Fig. 3 |
| G6 GSP 3` | 5-CAGCACCCAAGAAATCACAA-3 | Genomic | Fig. 3 |
| Neo 5` | 5-CGTTCCTTGCGCAGC-3 | Genomic | Fig. 3 |
| Neo 3` | 5-GGTGGTCGAATGGGC-3 | Genomic | Fig. 3 |
| Neo to OF 5` | 5-CTTCTTGACGAGTTCTTCTGAG-3 | Genomic | Fig. 3 |
| Neo to OF 3` | 5-GGTATTGTTAGCGAAATTGAAAGAA-3 | Genomic | Fig. 3 |
| RPL21 5` | 5-AAGTTGGTTATCAACTGTTGCGTT-3 | Genomic | Fig. 3 |
| RPL21 3` | 5- GGGTCTTTCAAGGACGACGTA-3 | Genomic | Fig. 3 |
| G6 GSP1 5` | 5-CTTGTGATTTCTTGGGTGCTG-3 | cDNA | Fig. 4 |
| G6 GSP1 3` | 5-CCATGAAATAATCAATATAGAGGGATA-3 | cDNA | Fig. 4 |
| G6 GSP2 5` | 5-CATGGATTTGGATTATGTTTGAAAG-3 | cDNA | Fig. 4 |
| G6 GSP2 3` | 5-TCACATGTTTCTCCTTCTTCA-3 | cDNA | Fig. 4 |
| RPL21-1 5` | 5-GGCTGTTCACGTTACCATC-3 | cDNA | Fig. 4 |
| RPL21-1 3` | 5-GGGTCTTTCAAGGACGAC-3 | cDNA | Fig. 4 |
