# Supplementary figures and images for: A Knockout Mutation of a Constitutive GPCR in Tetrahymena Decreases Both G-Protein Activity and Chemoattraction
Source: PLoS One. 2011 Nov 29;6(11):e28022. doi: 10.1371/journal.pone.0028022 (PMC3226668; doi:10.1371/journal.pone.0028022)

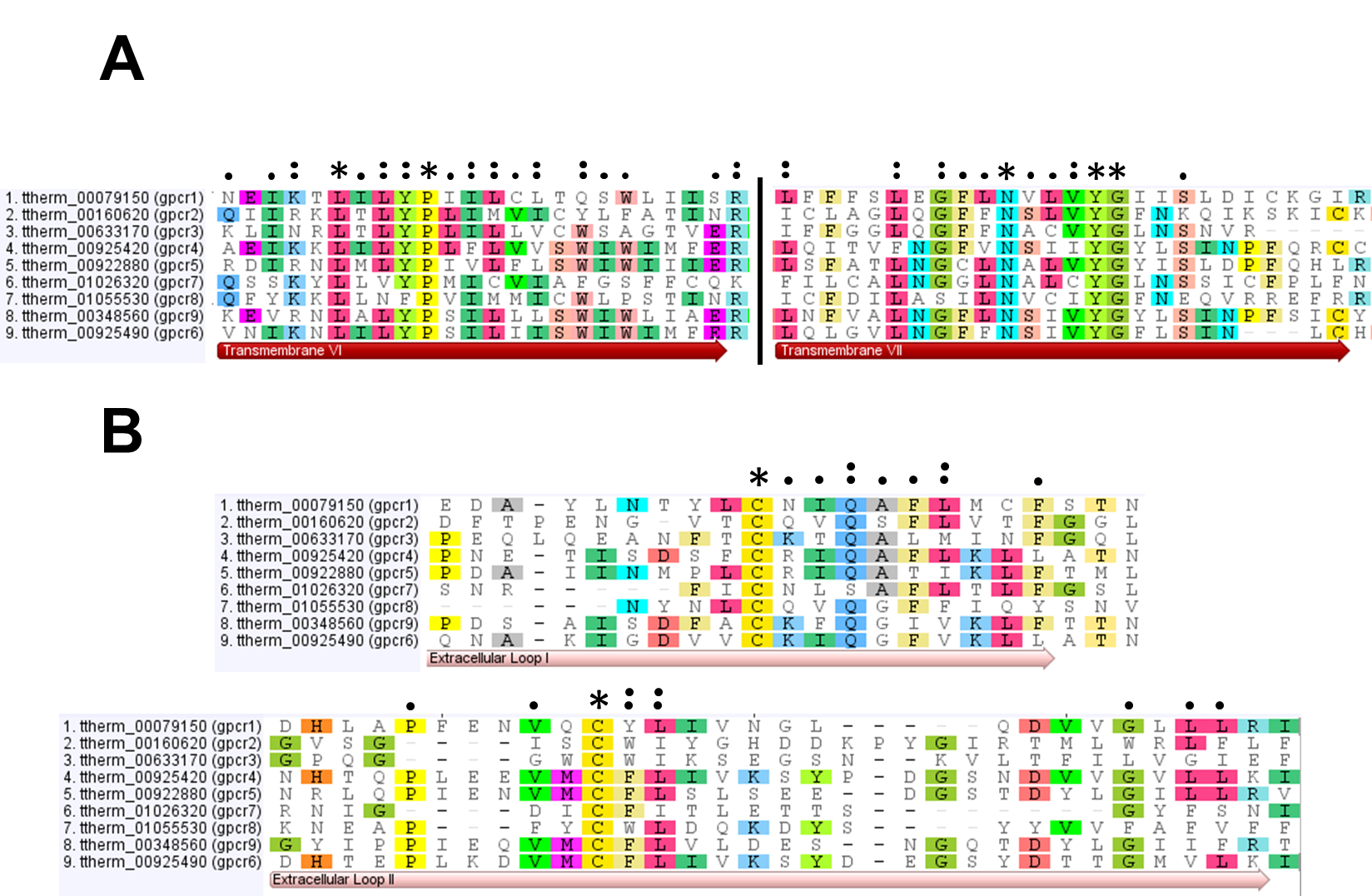

Supplement: Figure S2 — Conserved domains and residues in Tetrahymena GPCRs. A. ClustalW alignment between all 9 Tetrahymena GPCRs for transmembrane domains VI/VII. The Tetrahymena Gpcr6p and Arabidopsis gcr1 homologous transmembrane regions VI and VII share strong homology across all Tetrahymena GPCRs. B. A common GPCR conserved cysteine residue is seen in extracellular loops I and II in all predicted Tetrahymena GPCRs. A star depicts completely conserved residues (100% similar), double dot signifies high similarity (80–100% similar), whereas a single dot represents partial conservation (60–80% similar). (DOC) [file pone.0028022.s002.doc]

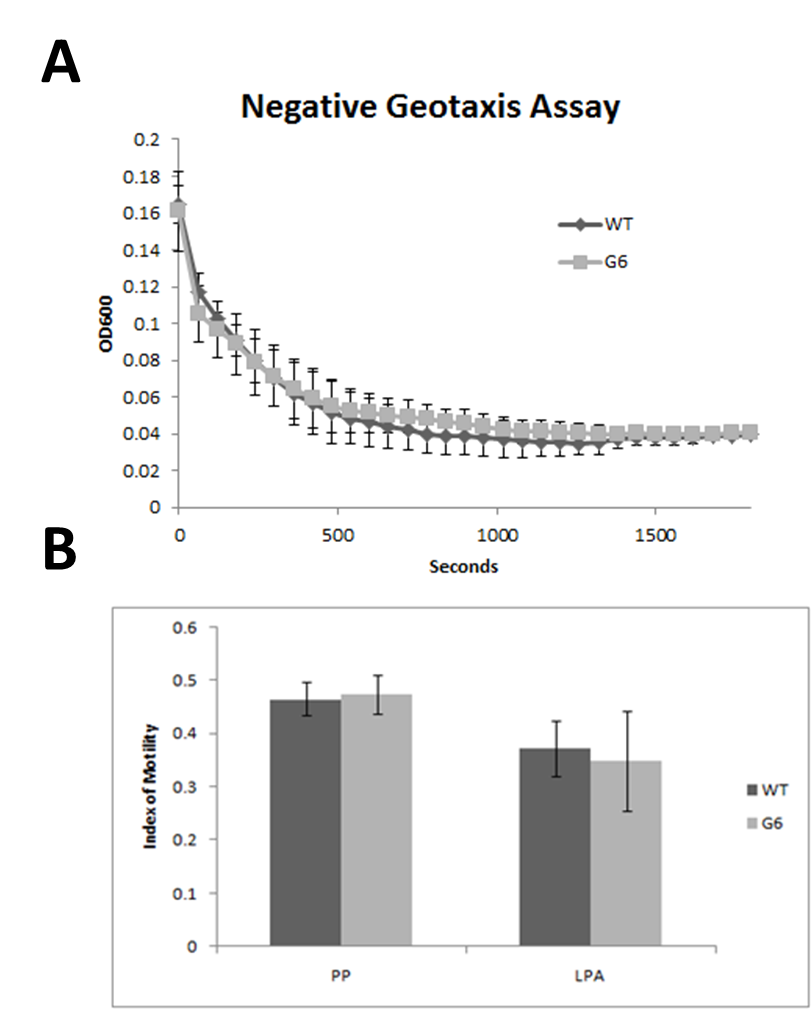

Supplement: Figure S3 — Chemoattraction control experiments. A. By placing cells in the cuvette alone (no attractant) the rate at which they rise to the top of the solution can be reflected by a decrease in absorbance. Both wild-type (WT) and G6 cell types have the same rate of negative geotaxis (n = 3). B. The index of motility, the number of cells that moves into the experimental region of the three-way stop cock assay, is the same in the G6 mutant. (DOC) [file pone.0028022.s003.doc]
